# Targeted Delivery of Zinc Pyrithione to Skin Epithelia

**DOI:** 10.3390/ijms22189730

**Published:** 2021-09-08

**Authors:** Sean E. Mangion, Amy M. Holmes, Michael S. Roberts

**Affiliations:** 1 Therapeutics Research Centre, Basil Hetzel Institute for Translational Health Research, The Queen Elizabeth Hospital, Woodville, SA 5011, Australia; sean.mangion@mymail.unisa.edu.au (S.E.M.); amy.holmes@unisa.edu.au (A.M.H.); 2 UniSA: Clinical and Health Sciences, University of South Australia, Adelaide, SA 5000, Australia; 3 Sydney Medical School, University of Sydney, Camperdown, NSW 2050, Australia; 4 Therapeutics Research Centre, Diamantina Institute, Translational Research Institute, University of Queensland, Woolloongabba, QLD 4102, Australia

**Keywords:** seborrheic dermatitis, dandruff, *Malassezia* yeast, toxicology, fluorescent lifetime imaging microscopy

## Abstract

Zinc pyrithione (ZnPT) is an anti-fungal drug delivered as a microparticle to skin epithelia. It is one of the most widely used ingredients worldwide in medicated shampoo for treating dandruff and seborrheic dermatitis (SD), a disorder with symptoms that include skin flaking, erythema and pruritus. SD is a multi-factorial disease driven by microbiol dysbiosis, primarily involving *Malassezia* yeast. Anti-fungal activity of ZnPT depends on the cutaneous availability of bioactive monomeric molecular species, occurring upon particle dissolution. The success of ZnPT as a topical therapeutic is underscored by the way it balances treatment efficacy with formulation safety. This review demonstrates how ZnPT achieves this balance, by integrating the current understanding of SD pathogenesis with an up-to-date analysis of ZnPT pharmacology, therapeutics and toxicology. ZnPT has anti-fungal activity with an average in vitro minimum inhibitory concentration of 10–15 ppm against the most abundant scalp skin *Malassezia* species (*Malassezia globosa* and *Malassezia restrica*). Efficacy is dependent on the targeted delivery of ZnPT to the skin sites where these yeasts reside, including the scalp surface and hair follicle infundibulum. Imaging and quantitative analysis tools have been fundamental for critically evaluating the therapeutic performance and safety of topical ZnPT formulations. Toxicologic investigations have focused on understanding the risk of local and systemic adverse effects following exposure from percutaneous penetration. Future research is expected to yield further advances in ZnPT formulations for SD and also include re-purposing towards a range of other dermatologic applications, which is likely to have significant clinical impact.

## 1. Introduction

**Figure 1:**
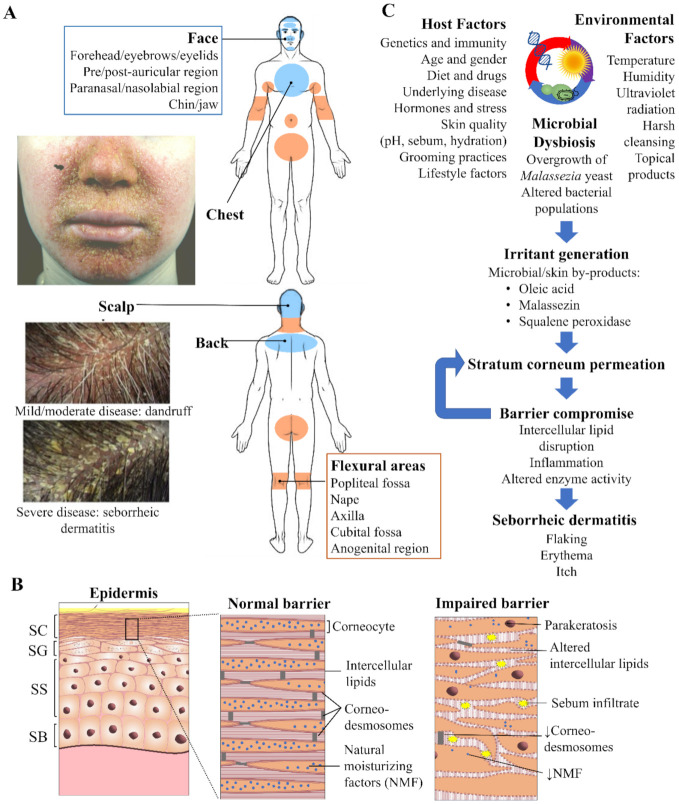
Seborrheic dermatitis and the skin. (**A**) Overview of the distribution pattern of seborrheic dermatitis with accompanying clinical photographs of selected regions. Veraldi et al. (2020), reproduced with permission [14] and Schwartz et al. (2015), reproduced with permission [15]. (**B**) Epidermal structure highlighting key features of the normal stratum corneum (SC) barrier and impaired barrier in seborrheic dermatitis. Proliferation of keratinocytes occurs exclusively from the stratum basale (SB). As these cells progressively travel upwards differentiation occurs in the stratum spinosum (SS) and granulosum (SG), resulting in the cornified cells of the SC, which are continuously shed from the skin surface by the process of desquamation. (**C**) Flow schematic demonstrating the interplay of microbial, host and environmental factors that drive seborrheic dermatitis pathogenesis.

Zinc pyrithione (ZnPT) is a widely used anti-fungal ingredient in shampoo indicated for treating the symptoms of seborrheic dermatitis (SD) and dandruff. It has led a profitable history within the personal care industry, demonstrating how ongoing research efforts are improving topical formulation for the consumer market. Dandruff is estimated to affect 50% of the population [1], producing scalp flaking, pruritis and erythema [2,3], all of which can significantly impact daily living and psycho-social wellbeing [4,5]. In the more severe form it presents as SD, a cutaneous disorder that affects approximately 1–3% of the adult population [6,7], resulting in more clinically significant scaling and irritation that can occur beyond the scalp (including the face, chest, back and flexural surfaces, Figure 1A) [8]. ZnPT was first synthesized during an anti-microbial screening program in the 1950s, originally established for agricultural purposes [9], and has remained a key commercial ingredient in the haircare industry since the 1960s after it was identified as a candidate anti-dandruff agent [10]. It is now the most popular anti-dandruff ingredient, listed in over 100 different topical products across multiple countries [11], at concentrations up to 2% *w*/*v* in rinse-off formulations [12]. While ZnPT can be incorporated into different formulations including lotions and tonics, shampoos are the most convenient and patient friendly. Therapeutic shampoos, such as those containing ZnPT, attract approximately 512 million USD of consumer expenditure annually in the Unites States alone [13].

Anti-dandruff shampoos are one of the few over-the-counter topical products regulated both as a drug, where treating SD and dandruff is concerned, and cosmetic, due to the cleansing function of the shampoo [16]. ZnPT shampoos are continuously developed and strongly marketed, especially towards formulations that act faster and have multi-functional benefits. Numerous clinical studies demonstrate efficacy of ZnPT shampoo against the signs and symptoms of dandruff and SD [17,18,19,20,21]. This review will explore the interactions of ZnPT with skin epithelia, imperative for both therapeutic efficacy and safety.

## 2. Skin Barrier Disturbance in Seborrheic Dermatitis

Normal barrier integrity of the epidermis (Figure 1B) is regulated through the coordinated events of keratinocyte proliferation, differentiation, and desquamation. Electron microscopy of the SD affected stratum corneum has revealed disordered ‘bricks and mortar’ packing as well as excess lipid accumulation [22]. Skin contains lipid of two origins differing in both composition and purpose [23]. Lipids associated with the stratum corneum are synthesized during keratinocyte differentiation within intracellular lamellar bodies and upon extrusion create lipid bilayers with a highly regulated packing arrangement, ranging from fluid to crystalline [24]. The intercellular lipids represent the ‘mortar’ between the corneocyte ‘bricks’ and are essential for the epidermal water barrier and mechanical properties of the stratum corneum [25,26]. In SD the amounts of intercellular lipids are reduced and relative ratios are altered [27], supportive of changes to their packing arrangement.

The second type of lipid, sebum, originates from the exocrine secretions of the sebaceous glands and associated hair follicle. It is believed that sebum may participate in facilitating desquamation [28]. Clinically, ‘dry’ and ‘oily’ dandruff have been described [29], however the latter may simply represent the more severe form of SD with increased sebum a compensatory protective response of the skin to excess dryness. These sebaceous lipids have been identified using in vivo Raman spectroscopy in the lower stratum corneum in SD [20], where they likely are capable of disrupting the normal intercellular lipid bilayer structure and contributing to excessive desquamation [30].

In SD the stratum corneum is functionally compromised, demonstrated by elevated trans-epidermal water loss (TEWL) [29,31]. The weakened barrier is characterized by increased rates of keratinocyte proliferation and immature corneocytes reaching the skin surface [32], as evidenced by parakeratosis and reduced desmosome density [22,33]. Inflammation is also a key feature with elevated histamine [18] (responsible for itch) and proinflammatory cytokines such as interleukin(IL)-1α, IL-1β, IL-2, IL-4, IL-6, IL-10, IL-12 and tumor necrosis factor-α [34,35,36]. Other epidermal changes include reduced expression of transglutaminase enzymes [37] involved in maturation of the cornified envelope [38] and reduced filaggrin proteins [37], which are precursors to natural moisturizing factors (NMFs) that contribute to skin flexibility [20,37].

Current evidence indicates that epidermal changes in SD arise from a complex interplay of microbial, host and environmental factors (Figure 1C). Previous work has highlighted the importance of different aetiologic factors in SD including stratum corneum dysfunction [39], *Malassezia* yeast and sebum [40,41] as well as the wider microbiota, including bacteria [42]. Advances are continuing to be made in understanding the relative importance and inter-relations of each of these factors [43,44,45,46].

## 3. Microbial Dysbiosis as a Key Target for Treatment

Sebaceous skin sites, particularly on the scalp, provide a rich microenvironment for the colonization of bacteria, mainly *Staphylococci* and *Propionibacterium* species, as well as *Malassezia* yeast species [47]. The healthy cutaneous microbiome forms a symbiotic relationship with the host capable of supporting immunity [48], for example through competitive colonization analogous to the gut [49], upregulation of anti-microbial peptides [50] and promotion of compliment [51]. In dysbiosis the balance of micro-organisms is altered, driving skin disorders such as dandruff and SD [43,45].

As early as 1874 lipid-dependent *Malessezia* yeast were observed colonizing scaly lesions on the scalp [52]. Several lines of evidence followed linking *Malassezia* to SD symptomology. Firstly, *Malessezia* load correlated with condition severity [53], secondly, symptoms could be induced by *Malessezia* inoculation in guinea pig [54,55] and thirdly, treatment occurred with a range of anti-fungal agents from zinc and selenium salts to azoles [54,56,57,58,59,60]. An excellent review of the early evidence is explored by Shuster [57].

*Malessezia* yeast have fastidious culture requirements and have had several nomenclature changes [61], with 18 species now recognized [62]. *Malassezia globosa* and *Malessezia restricta* predominate on the scalp [63,64], where they secrete lipases to breakdown sebum into fatty acids that can be assimilated for growth and survival [65,66]. A range of skin irritants have been implicated from the metabolic by-products of this breakdown, including oleic acid, malassezin, pityriacitrin, indolocarbazole and squalene peroxide [40,67,68,69]. These by-products can partition into the stratum corneum, disrupt normal lipid bilayer structure [40,70] and trigger an irritation response that over time is believed to result in a pattern of structural (altered lipid and corneocyte packing), functional (elevated TEWL, altered pH and sebum secretion) and clinical features (flaking, erythema and itch) consistent with dandruff and SD [71,72]. IL-17, IL-4, IL-8 produced by immune cells such as neutrophils and lymphocytes, as well as keratinocytes themselves (via Toll-like receptor stimulation) are involved in inflammatory signaling that may link these events and lead to progression and maintenance of impaired barrier function [73].

Understanding the nature of microbial perturbations in SD is still an active area of research. It should be noted that studies of microbial load and SD severity have produced conflicting data in the past. While some reports demonstrated a correlation [53], others failed to demonstrate any relationship [74]. Fastidious culture requirements and the multi-dimensionality now recognized for SD pathogenesis are potential contributing factors. There are also contrary findings about the ability of *Malassezia* to assimilate unsaturated fatty acids for growth [75,76], which has implications for elucidating the specific pathways underlying SD pathogenesis. Furthermore, while *Malassezia* yeast, particularly *M. globosa* and *restricta* likely play a key role, emerging data is also pointing towards involvement of bacteria, including an increase in *Staphylococcus* and a decrease in *Propionibacterium* species [43].

Although the picture of microbial dysbiosis is currently incomplete (particularly whether it is a cause or consequence of disease [77]), work thus far indicates that microbe biodiversity and abundance, influenced by host or intrinsic factors (e.g., age, gender, immunity and hormonal status) [78,79] and environmental or extrinsic factors (e.g., climate, physical activity, diet, grooming products or even face mask wearing as highlighted during the recent COVID-19 pandemic) [14,80], are important in maintaining skin health or driving disease. The microbial component of disease has therefore been an important target in treatment with anti-fungal therapies such as the commercially popular ZnPT available over-the-counter. Indeed, histologic, biomolecular, and biophysical investigations demonstrate that clinical symptom improvement after treatment with ZnPT parallels restoration of stratum corneum structure and function, as well as the quality of emergent hair fibers [20,22,36,81,82,83].

## 4. Zinc Pyrithione (ZnPT)

### 4.1. Zinc Pyrithione Structure and Physicochemical Properties

**Figure 2:**
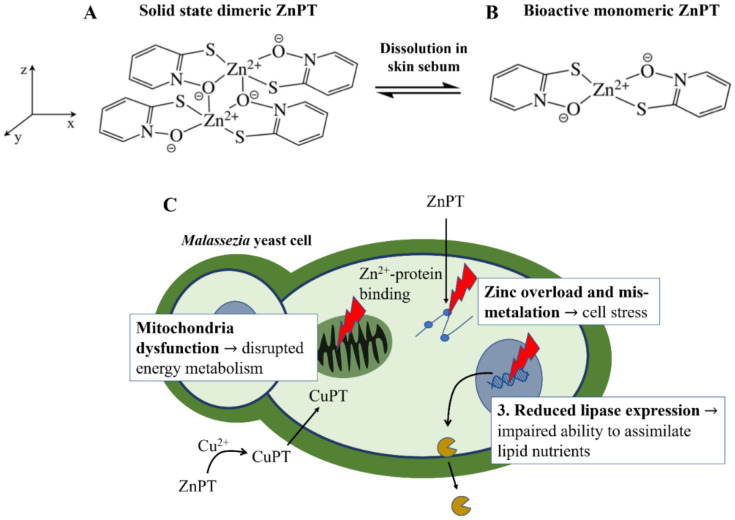
Zinc pyrithione molecular structure and anti-fungal mechanisms of action. (**A**) The dimeric structure of ZnPT in the solid state and (**B**) after dissolution into the bioactive form. (**C**) The three key actions of ZnPT on *Malassezia* yeast, resulting in zinc-induced cellular stress, disrupted energy metabolism and impaired ability to assimilate lipid nutrients for growth.

ZnPT is a solid coordination complex of zinc with a molecular weight = 317.7 g/mol and logP = 0.88. These properties would ordinarily render a molecule highly permeable when applied on the skin, however skin permeation is limited for ZnPT due to low aqueous solubility of 5–15 ppm [12]. The rigid crystal lattice structure exists in the solid-state where two bridging oxygens form a pseudo-dimer between two ZnPT molecules (Figure 2A) [84]. Aside from limiting its permeability, the sparse water solubility also makes ZnPT ideal for deposition on the skin from rinse-off formulations, particularly in lipid rich regions such as the scalp. As ZnPT particles gradually dissolve the bioactive monomeric form (Figure 2B) is liberated, a structural analogue of the natural antibiotic aspergillic acid with anti-fungal activity against *Malassezia* yeast [85].

### 4.2. Anti-Fungal Mechanisms of Action

ZnPT has several mechanisms of action shown in Figure 2C. Firstly, as pyrithione is a known zinc ionophore, ZnPT causes elevated intracellular zinc levels in yeast cells [85,86] to the extent that it results in mismetallation and cellular stress [87]. Secondly, endogenous levels of copper, which may be released during skin renewal or supplied by the immune system, are also involved in growth inhibition [88]. Anti-fungal effects are thought to occur via an extracellular transchelation reaction converting ZnPT into copper pyrithione, resulting in microbial intracellular copper influx [88,89]. This excess intracellular copper can then inactivate aconitase, which is an enzyme involved in fungal energy production within the mitochondria [90]. Studies have also demonstrated ZnPT inhibits the membrane transport of nutrients needed for yeast growth by inducing membrane depolarization [91,92,93,94]. Lastly, relatively recent evidence has demonstrated that in *Malassezia restricta* ZnPT can reduce lipase expression, critical for lipid breakdown, as well as downregulate gene expression for components of the Krebs Cycle (e.g., succinate dehydrogenase and citrate synthase) and electron transport chain (e.g., ATP synthase subunit) [86]. Together, these studies suggest ZnPT impairs the ability of fungal cells to assimilate and metabolize nutrients for growth, leading to growth inhibition. The relative contributions of each of these mechanisms to overall activity is not known, however the fact that ZnPT acts via multiple pathways likely explains why fungal resistance to this agent has not to our knowledge been reported (although resistance in *Pseudomonas* bacteria has been [95]).

### 4.3. Pharmacological Response: Anti-Fungal Susceptibility Testing to Determine Target Doses for Topical Delivery

**Figure 3:**
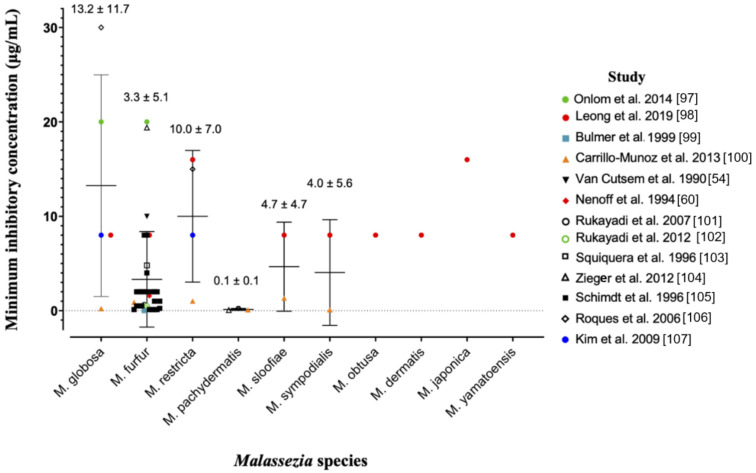
Pooled analysis of minimum inhibitory concentrations for zinc pyrithione against various species of *Malassezia* yeast. Individual studies are coded in the legend. Where the MIC of the original dataset was reported as a range, only the upper limit was included as a conservative approach to analysis. Data is presented as mean ± standard deviation.

The anti-fungal potency of ZnPT has been widely studied in vitro using the gold-standard broth microdilution susceptibility test [96]. The minimum inhibitory concentration (MIC) is the lowest concentration of ZnPT that causes a decrease in yeast suspension turbidity, indicative of reduced fungal growth. Figure 3 presents a pooled analysis (mean ± standard deviation) of anti-fungal MIC values from multiple studies against different *Malassezia* species [54,60,97,98,99,100,101,102,103,104,105,106,107].

Prior work suggests that *Malassezia globosa* and *restrica* may be more susceptible compared to *Malassezia fufur* [98], however the pooled analysis presented here demonstrates the opposite trend. Intra-species MIC values range significantly for most species, for example from 0.21 µg/mL [100] to 30 µg/mL [106] for *Malassezia globosa*. Different strains and assay methods are likely contributors. The ZnPT preparation is also important for anti-fungal bioavailability, as particles are more soluble in certain solvents such as dimethyl sulfoxide (DMSO) (30 mg/mL) and ethanol (0.31 mg/mL) compared to water and saline (0.015 mg/mL) [12,108]. This can have a significant effect on MIC value, for instance when DMSO is substituted for distilled water in two similar studies [97,98] with the same strain of *Malassezia fufur* (CBS 1878) the MIC increases 2.5-fold from 8 µg/mL to 20 µg/mL. Studies have also demonstrated no difference in fungal susceptibility of *Malassezia globosa* from patients who use and do not regularly use anti-fungal shampoo, which is an important consideration for continued maintenance therapy [109]. Overall, our analysis reveals that for the two most important fungal species in dandruff and SD, *Malassezia globosa* and *Malassezia restrica* [63,64], average effective anti-fungal concentrations for topical delivery should be approximately 10–15 µg/mL.

A noteworthy limitation of the broth microdilution method for establishing an effective anti-fungal dose is that it is unable to capture the host-microbe interactions, which occur in vivo, that may modify fungal susceptibility to ZnPT. Further investigations using methods should be performed that can (1) better replicate conditions of the scalp micro-environment in a controllable system (that way removing the confounding variables that may occur in vivo) and (2) better replicate the exposure and contact that occurs after topical administration of ZnPT. A range of in vitro and in vivo methods for further exploring host-microbe interactions has recently been reviewed [110]. One interesting method that has been reported separately involves inoculation of hair strands in agar after simulated shampoo washing and rinsing [111].

There is also access to more sensitive yeast identification tools such as PCR determination, based on internal transcribed spacer regions of ribosomal DNA [112], which has been used to follow yeast loads over 7 days [109]. One limitation of this approach (compared to culture-based methods) is that it is not able to differentiate between live and dead yeast. A combination of culture-based and molecular methods and more advanced techniques such as matrix-assisted laser desorption ionization time-flight mass spectrometry (MALDI-TOFMS) [113] are likely to provide a complementary balance of sensitivity and specificity required for future analyses in the field.

### 4.4. Deposition of ZnPT on Human Skin Epithelia

Deposition of ZnPT is important because the particles retained on the scalp following shampoo rinsing have the longest exposure time and are the main sources of anti-fungal action [114,115]. For targeted anti-fungal delivery, deposition should mirror the spatial distribution of *Malassezia* yeast on the skin. There are two locations to consider, including the hair follicles and the interfollicular skin surface.

**Figure 4:**
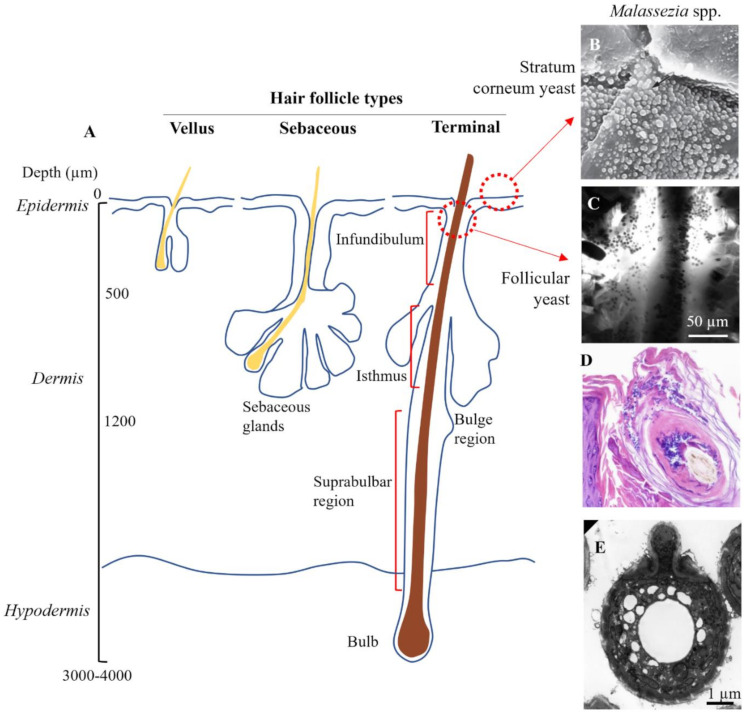
Target sites for topical zinc pyrithione. (**A**) Schematic showing anatomy of the three different types of hair follicle. (**B**) Surface colonization of Malassezia yeast (×2700 magnification). Piérard et al. (2006), reproduced with permission [117]. (**C**) Yeast colonization in the infundibulum imaged using in vivo confocal microscopy. Meyer et al. (2005), reproduced with permission [119]. (**D**) Yeast congregating and spreading within epithelial cells of the hair follicle (×20 magnification), reproduced from [124] in accordance with StatPearls Publishing LLC Creative Commons Attribution 4.0 International License. (**E**) Electron micrograph of Malassezia yeast isolated from the skin. Guého-Kellermann, et al. (2011), reproduced with permission [125].

Hair follicles are invaginations of the skin surface that support the hair in various stages of growth. The skin contains 3 distinct types of follicles, which vary in morphological characteristics and distribution (Figure 4A) [116]. Vellus follicles are the smallest, contain unpigmented hair and are found body wide, although predominate before puberty. Terminal follicles are significantly deeper, contain pigmented hairs and predominate at sites such as the scalp, axilla, pubic area, and the legs. Sebaceous follicles are moderately sized and contain numerous sebaceous glands, predominating on the face, back and chest. Terminal hair follicles are the most relevant to consider for scalp SD, while sebaceous follicles are the most relevant for SD occurring on the face, chest and back.

Buds of *Malassezia* yeast have been observed superficially in clumps on desquamating corneocytes [117] and interspersed between corneocyte layers of the stratum corneum, forming a particularly dense load in seborrheic dermatitis [22,118] (Figure 4B). *Malassezia* yeast have also been directly observed accumulating at follicle sites on the scalp (Figure 4C,D) [119]. While precise data on the follicle distribution of *Malassezia* is missing [120], the funnel shaped infundibulum at the uppermost region is an ideal site to act as a microbial reservoir as it provides protection against skin sloughing and is irrigated with sebaceous lipids. The infundibulum has also been postulated to have a more permeable stratum corneum with a higher density of antigen presenting cells in the lower region [121,122] which, if correct, could mean could that infundibular skin is at increased susceptibility to *Malassezia*-derived irritants and the subsequent inflammatory cascade. As well yeast, approximately 25% of skin bacteria in the human forearm are localized within the follicles [123].

**Figure 5:**
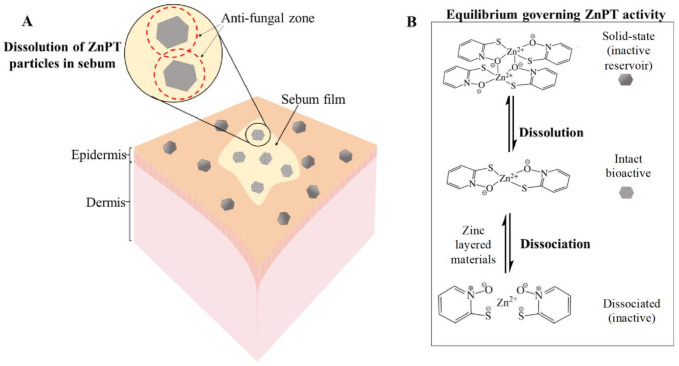
Zinc pyrithione deposition on the skin. (**A**) Solid-state ZnPT particle dissolution occurring in a film of sebum covering the skin surface releasing bioactive ZnPT. (**B**) Equilibria governing anti-fungal delivery to the follicles with zinc layered materials boosting levels of bioactive species.

The equilibria dynamics of ZnPT in the product packaging and once deposited onto the skin are important for governing anti-fungal bioactivity (Figure 5) [9,85]. ZnPT particles (solid state dimer) exist in an equilibrium with the dissolved state ZnPT (monomer), the latter of which represents the bioactive species. Activity can be lost if the coordination complex dissociates further into its zinc and pyrithione moieties. Delivery should be optimized to prevent active-complex dissociation and bioavailability has been improved through the addition of zinc layered materials such as zinc carbonate. This strategy provides a sustained source of common zinc ions that act to shift the equilibrium towards the bioactive complex, preventing dissociation in both the bottle and once deposited onto the skin. Shampoos containing additional zinc display enhanced anti-fungal activity in vitro against *Malassezia globosa*, as well as improved anti-dandruff efficacy in vivo [56].

## 5. Skin Imaging Methods to Evaluate the Spatial Delivery of ZnPT

A range of complementary imaging methods have been used over the past 50 years for measuring the delivery of ZnPT after topical application to the skin [56,85,115,126,127,128,129,130,131,132]. Each modality carries advantages and disadvantages summarized in Table 1. Factors such as study design (in vivo realistic exposure or ex vivo controlled dosing), sample preparation (physical or optical sectioning) and type of skin (human or animal) are potential confounders in interpreting the results of imaging data.

### 5.1. Autoradiography

**Figure 6:**
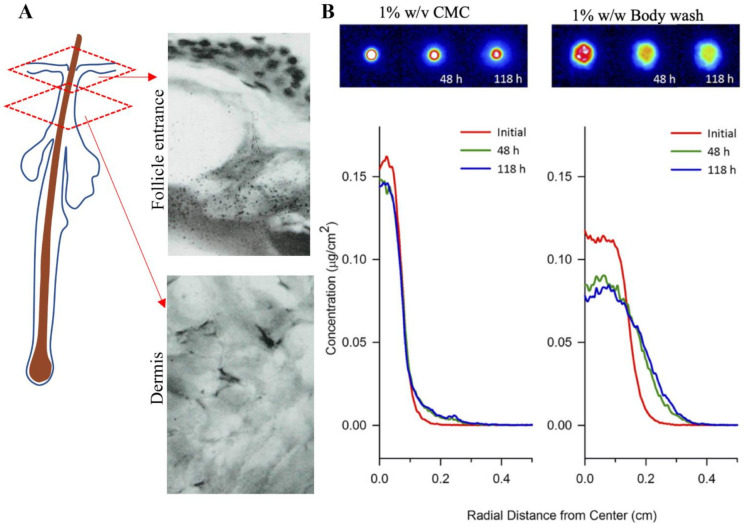
Autoradiography for assessing zinc pyrithione topical delivery. (**A**) Radiograph of lateral cryosection demonstrating moderate grain density of ZnPT particles localized around the entrance of a follicle (upper panel), but not in the dermis (lower panel) of guinea pig skin (×500 objective). Rutherford et al. (1969), reproduced with permission [126]. (**B**) False-colored quantitative radioluminographic images (upper panels) with accompanying average concentration-distance profiles (lower panels) for initial and subsequent ZnPT dose distributions on human skin, demonstrating greater lateral micro-transport of ZnPT with time in 1% body wash *w*/*w* (right) compared to aqueous carrier containing 1% *w*/*v* carboxymethylcellulose (CMC, left). Rush et al. (2015), reproduced with permission [127].

Autoradiography was the earliest method of imaging ZnPT on skin [126]. Radiographs of lateral cryosections of guinea pig skin dosed with [^35^S]-labelled ZnPT shampoo (1.7% *w*/*v*) showed accumulation around the follicle entrance with no dermal penetration (Figure 6A). Similar principles have been used recently in quantitative radioluminographic imaging to calculate kinetics of ZnPT micro-transport across dermatomed human skin (Figure 6B) [127]. Results show a greater lateral diffusivity of ZnPT in 1% *w*/*w* body wash (9.2 × 10^−9^ cm^2^/s) compared to 1% *w*/*v* carboxymethylcellulose formulation (0.94 × 10 10^−9^ cm^2^/s). These formulation effects are an important factor for anti-fungal bioavailability of ZnPT to target structures in the skin.

### 5.2. Confocal Microscopy

**Figure 7:**
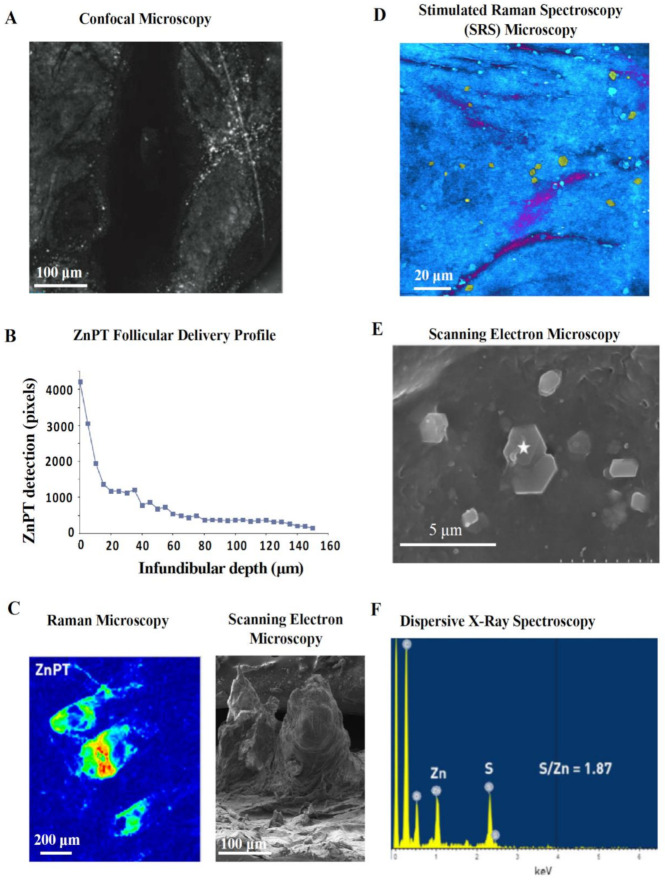
Reflectance and scattering methods for assessing zinc pyrithione topical delivery. (**A**) In vivo reflectance confocal image (40 μm) of human hair follicle showing particle deposition and (**B**) accompanying pixel-depth profile. Schwartz et al. (2011), reproduced with permission [115]. (**C**) Raman microscopy image, intensity weighted by color (red—high, blue—low), of an ex vivo cyano-acrylate hair follicle biopsy with accompanying scanning electron microscopy (SEM) image of cyano-acrylate follicular cast. Chen et al. (2017), reproduced with permission [128]. (**D**) Stimulated Raman Spectroscopy (SRS) microscopy image of ZnPT (yellow particles) and climbazole (magenta) on the surface of ex vivo porcine skin. Garrett et al. (2017), reproduced with permission [129]. (**E**) SEM image of tape stripped skin from scalp washed with ZnPT shampoo with (**F**) accompanying dispersive X-ray spectra (from the star marked region) confirming its chemical identity. Chen et al. (2018), reproduced with permission [130].

Confocal microscopy is a safe and non-invasive tool that has been used in vivo to image ZnPT on skin [56,115]. Adjusting the focal plane in tissue allows optical sectioning after lather and rinsing of ZnPT shampoo. Reflectance confocal imaging shows ZnPT delivery to the follicular infundibulum [115] (Figure 7A) with greater resolution compared to initial radioisotope work (Figure 6A) [126]. The depth profile for delivery derived from pixel counting algorithms demonstrates that deposition predominates within the upper 40 μm of the infundibulum (Figure 7B) [115]. While there is signal detected up to 150 μm, a key disadvantage of this technique is that it lacks chemical specificity. Therefore, it is possible for skin debris and other ingredients deposited on the scalp, such as zinc carbamate [56,130], to be detected in the reflectance mode where there is no way of discriminating these signals from ZnPT.

### 5.3. Raman Spectroscopy

Chemical specificity is an advantage of Raman microscopy, which relies on detecting the Raman scattering of a sample after infrared excitation to determine molecular vibrational modes [133]. ZnPT can be discriminated based on signals from chemical motifs not present in human skin or other ingredients [134]. Raman imaging has been used ex vivo on human cyanoacrylate glue casts collected after shampoo application to visualize the follicular deposition of ZnPT with relative signal intensity mapping (Figure 7C) [128]. While traditional Raman offers low spatial resolution, Stimulated Raman Scattering (SRS) microscopy improves resolution by coherent excitation [135] and has been used on porcine skin to show ZnPT delivery on the scalp surface with very good visualization of particle morphology (Figure 7D) and follicular delivery up to 10–20 µm [129].

The difference in ZnPT follicular delivery reported using Raman [129] compared to reflectance confocal microscopy [115] could be explained by the weaker detection ability of Raman at greater tissue depth, formulation factors specific to the shampoo, or differences in ex vivo porcine skin and in vivo human skin—although if this was the case greater delivery might be expected with the larger follicles of porcine skin [136]. Lastly, the discrepancy in follicular delivery may be due to interference in reflectance confocal images occurring as a result of low chemical specificity.

### 5.4. Scanning Electron Microscopy

Scanning electron microscopy (SEM) enables high resolution and magnification of ZnPT, especially suited for assessing morphology of particles deposited on the scalp (Figure 7E). Coupling SEM with dispersive X-ray spectroscopy of tape strips from scalp skin washed with ZnPT shampoo allows separate identification of ZnPT and zinc carbamate particles based on characteristic signals from zinc and sulfur (Figure 7F) [130]. This method has been useful for investigating the influence of particle size and shape on ZnPT retention, demonstrating that 2 μm plate shaped particles are optimal, at least for scalp surface delivery, which is consistent with earlier reports [85].

### 5.5. Fluorescence Lifetime Imaging Microscopy

The imaging methods discussed thus far have been primarily limited either by spatial resolution, chemical specificity, or a combination of both. We have previously reported and characterized the optical properties of ZnPT using both single-photon and two-photon laser excitation [131], which has opened up the opportunity of using new imaging methods for sensitive and specific ZnPT skin delivery assessment both ex vivo and in vivo. Fluorescent lifetime imaging microscopy (FLIM) is an ideal tool that can resolve images both spectrally and by fluorophore lifetime, thereby enabling high chemical sensitivity [137].

**Figure 8:**
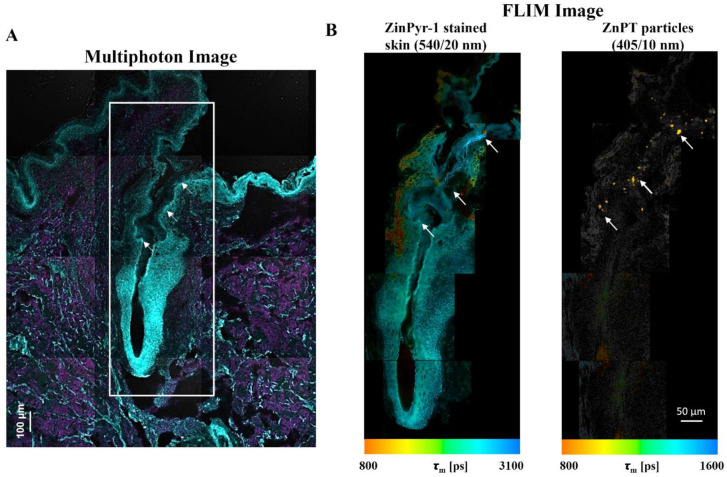
Fluorescence lifetime imaging microscopy (FLIM) for assessing zinc pyrithione follicular delivery to human skin. Skin was dosed with 2% *w*/*v* aqueous ZnPT suspension and cryosectioned to 20 µm. (**A**) ZinPyr-1 stained skin imaged with multiphoton microscopy (two-photon excitation, cyan region λ_ex_ = 800 nm, λ_em_ = 370–420 nm; single-photon excitation, magenta region λ_ex_ = 488 nm, λ_em_ = 520–560 nm). (**B**) FLIM image pseudo-coloured to average time-weighted lifetime, τ[picoseconds] (two-photon excitation; λ_ex_ = 740 nm; emission detected using two bandpass filters indicated). Arrows mark ZnPT particle deposition.

With the methods previously described [131], we have used multiphoton microscopy (MPM, Figure 8A) combined with FLIM (Figure 8B) to map ZnPT delivery within the hair follicles of human skin [132]. ZinPyr-1 is a fluorescent probe used to detect labile zinc within tissue and it has previously been applied to observe relative increases in labile zinc within skin after topical application of zinc species [138,139,140]. Staining with ZinPyr-1, enables visualization of increased labile zinc within the skin while the inherent luminescence of ZnPT enables visualization of particle deposition. Using MPM-FLIM we observed delivery of ZnPT from a 2% *w*/*v* aqueous suspension up to approximately 200 μm within the hair follicle. Imaging with FLIM will be important in future studies to explore delivery from commercial ZnPT formulations.

### 5.6. Radiolabeling Studies to Quantify Exposure

Imaging studies provide spatial information and topical application, however rarely can provide accurate quantitative data on delivery amounts. Quantification of dose can be performed with a range of chromatography, spectroscopy and radiolabeling methods highlighted in Table 2 [12,115,128,130,141,142,143,144]. An important consideration for formulation optimization is that the same quantitative approach must be followed when comparing delivery between formulations, as each analysis method will have different levels of accuracy and precision.

Much like imaging studies, radiolabeling was the earliest approach used for quantifying delivery. After application of [^35^S] labelled ZnPT to the forearm in human subjects, Geiger counting demonstrated that approximately 1% of the total applied ZnPT dose remains on the skin after shampoo rinsing [144]. Scintillation counting has been performed with human, rat and monkey skin following tape strip collection to determine the degree of the stratum corneum ZnPT reservoir, with the amount ranging from 0.0035 μg/cm^2^ (0.1% *w*/*v* formulation on monkey skin) [143] up to 48.94 μg/cm^2^ (48% *w*/*v* formulation on rat skin) [12] depending on the formulation, skin and number of tape strips removed. Interestingly, using analysis of hair clippings approximately 5-fold more ZnPT was found to deposit on the hair fiber itself [12], with a clinical significance that is unknown and requires further investigation (this perhaps may define a third target site along with the scalp surface and hair follicle).

## 6. Toxicity Assessment

Safety is a concern that must always be balanced with efficacy. To quote the father of toxicology, Paracelsus (1493–1541 AD), ‘*it is the dose that distinguishes poison from remedy*’. In order to understand the safety profile of topical ZnPT it is important to determine the exposure levels that cause toxicity, from non-lethal adverse effects to death, and then compare these to exposure levels that occur from product use. 

ZnPT has a long track record of safe use supported by independent regulatory evaluations such as the European Commission on the Scientific Committee of Consumer Safety [12]. Much of the toxicity data generated by industry is made available through these regulatory safety reports. It is considered safe in rinse-off formulations up to a maximum concentration of 2% *w*/*v* and in leave-on products up to 0.25% *w*/*v*. The following section will provide a discussion of the approaches that have been used in assessing safety, including studies investigating local and systemic toxicity.

### 6.1. Local Cutaneous Toxicity of ZnPT to Human Skin Epithelia

ZnPT has been assessed in both human and animal studies to determine local toxicity. This is distinct from systemic toxicity as it occurs at the site of application. It may manifest in different forms either as an acute or chronic reaction, including allergic and irritant contact dermatitis, photosensitivity, pigmentary changes (hypo- or hyper-pigmentation), hair changes (alopecia or hypertrichosis), drug eruptions (e.g., toxic epidermal necrolysis) or tumor development [146].

Although skin irritation and sensitization can bring about similar skin symptoms (e.g., erythema and itch), it is important to make a distinction between them, especially for safety assessment. Irritation occurs due to direct contact and does not involve an immune response [147]. It typically involves disruption to the stratum corneum barrier over a period of time as occurs in the case of surfactant-induced irritation [148]. In contrast, sensitization occurs by an immune response and involves both an induction phase, which occurs upon initial contact, and an elicitation phase, which occurs during subsequent contact, generating an immune skin reaction sometimes within minutes of exposure [149].

ZnPT has been described as, at worst, a mild irritant and ‘very weak’ sensitizing agent [12]. In vitro studies such as the chorioallantoic membrane irritation (HET-CAM) assay have been performed demonstrating the threshold for slight irritation (in this case vessel hemorrhage) to be 1 µM [150]. A limitation of this approach is that it poorly reflects realistic exposure on intact skin. More complex in vitro models exist using reconstructed skin, which have demonstrated a slight reduction in cell viability for exposure to 0.01% *w*/*v* ZnPT, with poor solubility limiting testing at the maximum commercial concentration of 2% *w*/*v* [151]. There is a clear need for in vitro models to better simulate in-use scenarios, particularly in terms of the formulations applied, as excipients are not inert as was once thought.

Human and animal in vivo studies better replicate in-use conditions and are conducted by exposing a defined skin area to the compound, sometimes under an occlusive or semi-occlusive patch, and observer grading of the skin response [152]. Bio-engineering tools such as trans-epidermal water loss and laser Doppler may also be employed in assessment [153]. ZnPT irritation testing has been conducted at a range of concentrations (up to 48% *w*/*v* in animals and 10% *w*/*v* in humans) under different patch conditions (open, semi-occlusive, and closed) and exposure times, including repeat insult up to several days. ZnPT demonstrates only mild skin irritation, even under exaggerated conditions [12].

In animal sensitization studies, which involve initial contact and subsequent challenge to simulate the elicitation and induction phases of response [154], ZnPT was deemed not to have any sensitization potential [12]. Unsurprisingly, for a drug so widely used since it was first approved there have been isolated case reports of human sensitivity to ZnPT [155,156,157,158,159]. The occurrence of sensitivity does however remain very low, with a positive patch test rate ranging from 0.002% (3 individuals out of 1652) [155] to 1.2% (2 individuals out of 171) [156]. In those who have suspected sensitization, testing using sodium pyrithione [160] suggests that the response may be due to the pyrithione moiety rather than the inorganic zinc moiety. The shampoo vehicle itself containing surfactants, preservative and fragrance likely poses a greater risk than the active material per se.

In regard to tumorigenic potential, lifetime studies in rats and mice have been conducted and demonstrate no evidence of carcinogenic potential from dermal doses up to 100 mg/kg/day [12]. ZnPT has also not been shown to cause pigmentary changes in humans from 2-month daily exposure at 0.2–2.0% under non-occlusive conditions [12].

Overall, evidence to date provides a strong basis for the safe use ZnPT in shampoo for treating SD. It is important to consider however that this evidence has focused primarily on detecting overt skin changes after exaggerated exposure for typically several days up to several months. Traditional methods that rely on observer scoring under these conditions are unable to detect subtle alterations due to sub-clinical levels of toxicity that may accumulate over time to cause effect. Sub-clinical toxicity my manifest as cellular changes, including changes in the redox state or induction of heat shock response gene expression. This has led to the description of the ‘invisible dermatoses’ by Kligman [161]. Emerging data combining advanced approaches to toxicological assessment is providing new insight into the potential of ZnPT to cause acute cellular zinc toxicity (even in nanomolar concentrations of ZnPT as shown by Lamore [162]) with a translational impact for human safety that is not yet known.

Zinc is essential for cellular health however only within a narrow concentration range, out of which, it becomes cytotoxic [163]. Studies using human keratinocytes demonstrate very high sensitivity to ZnPT (TD_50_ = 500 nM) at concentrations which are 100,000-fold lower than the 2% maximum level currently approved in shampoo [164]. Data from ex vivo static diffusion cell experiments reveal that viable epidermis and dermis may be exposed to ZnPT levels within this range (0.75 μg/cm^2^ for human skin) after topical exposure (Table 3) [12,115,128,141,142,143,145]. It should be noted however that this is based on results from Franz cell studies using [^14^C] ZnPT, which, as a radiolabel for the organic moiety, may not fully predict the levels of Zn^2+^ implicated in inducing cytotoxicity. While the organic moiety shows greater permeation into the circulation in the rat [165], it is unclear from this data whether dissociation occurs on the skin surface or within the viable skin, which is important for determining Zn^2+^ exposure.

**Figure 9:**
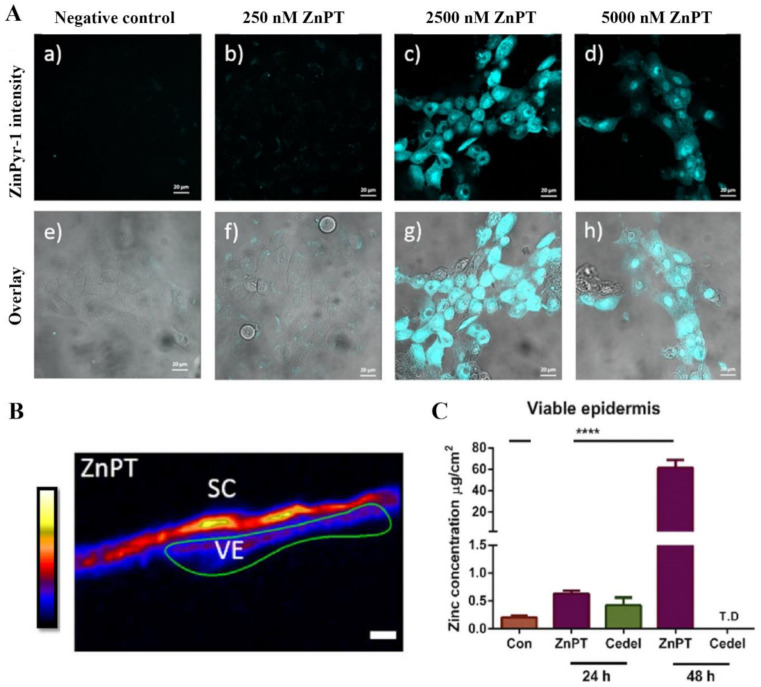
Zinc pyrithione effects on keratinocyte and human skin zinc levels. (**A**) Co-culturing HaCaT cells with ZnPT increases intracellular labile zinc. Confocal microscopy images of cells stained with ZinPyr-1 (10 μM), color coded cyan, after incubation for 24 h with various concentrations of ZnPT (0–5000 nM). Images displayed are representative of triplicate images, scale bar represents 20 μm. (**B**) Images of zinc distribution with XFM, zinc intensity ascribed an artificial colour range with black being zero concentration and white being the highest concentration. Scale bar represents 20 μm. (**C**) Zinc concentrations in the viable epidermis extracted from XRF images of zinc by selecting regions of interest. Holmes et al. (2018), reproduced with permission [138]. The **** represents *p* < 0.0001.

Our research group has used the labile zinc specific probe, ZinPyr-1, to show the intracellular zinc concentrations increase within keratinocytes in vitro when incubated with ZnPT (Figure 9) [138]. Furthermore, inductively coupled plasma mass spectrometry, expression array analysis and immunohistochemistry in re-constructed skin treated with ZnPT (0.1–2%) shows that keratinocyte toxicity may be mediated by increased influx of intracellular zinc, causing upregulation of heat shock protein, loss of genomic integrity and cellular energy crisis [162,166]. The question of whether similar processes occur in human skin has been partly addressed again by our group using synchrotron X-ray fluorescence, demonstrating that zinc concentrations significantly increase in ex vivo viable epidermis up to 3.8-fold after application of commercial 2% ZnPT formulation for 24 h (Figure 9) [138]. These results appear to demonstrate dissociation occurring primarily on the skin surface as opposed to the viable epidermis.

Overall, results suggest that ZnPT has the capability of over-riding the keratinocyte zinc homeostatic system. Further work is needed to determine whether hallmarks for cytotoxicity occur in human skin after application in realistic conditions and, if so, what the implications are for long-term exposure. This will require both gene and protein expression analysis as well as zinc concentration mapping from biopsies of human skin after such exposure. The extent to which Zn^2+^ can permeate into the underlying skin may be the key factor in regulating potential toxicity from this route. Further work should therefore also explore the sensitivities of different skin cells such as melanocytes or stem cell populations from the hair follicle bulge region to these effects.

### 6.2. Systemic Toxicity

Administration of ZnPT to different animal species (e.g., rat, mouse, dog and monkey) via different routes (oral, dermal, inhalation, peritoneal and intravenous) have been performed to establish toxic dose thresholds, i.e., the minimal dose that causes toxic effects. ZnPT toxicity is highly dependent on administration route. Inhalational and intravenous administration has the lowest threshold for toxicity, followed by intraperitoneal and oral administration [12]. Systemic effects range from emesis (oral administration in dog) [12], general cholinergic symptoms (intravenous administration in dog and monkey) [167], limb muscle weakness and lung swelling (inhalational administration in rat) to death [12,168,169]. Importantly, when ZnPT is administered via the skin in acute doses these symptoms do not occur [144,170]. The only acute symptoms reported are ‘slight temporary depression’ for doses on the order of 10–20 g/kg applied to abraded skin of restrained rabbits [144].

The threshold for toxic effects is lowered and the pattern of systemic effects is altered when ZnPT is provided in repeated doses over 2–13 weeks. Systemic symptoms include haematological changes and hind limb muscle atrophy or paralysis [12,144,171]. Hind limb functional effects were reversible, however electrophysiological dysfunction was still detectable [172]. Atrophy is reportedly due to muscle disuse, secondary to neurological effects [173,174], specifically involving peripheral axonopathy which in vitro studies indicate may be mediated by neuronal calcium influx [175,176].

The occurrence of hind limb weakness has been used as the critical effect for threshold exposure values in safety assessment, although the relevance of this to human toxicity is not known. A lethal topical dose has been reported as 1000–2000 mg/kg when applied 5 days per week for 90 days [12]. At lower doses of 100 mg/kg over 2 weeks hind limb weakness and local irritation occurs. For chronic dosing at 5 mg/kg/day for 80 weeks in rats, only local skin irritation has been reported [12].

### 6.3. Systemic Exposure Following Topical Application

Radiolabeling studies and more recently studies using liquid chromatography mass spectrometry [177] have been performed to determine the systemic exposure of ZnPT after topical application. The organic (pyrithione) and inorganic (zinc) moieties of ZnPT can be differentially labelled using isotopes of [^14^C] or [^35^S] and [^65^Zn], respectively. Dosing in rabbits has demonstrated that ZnPT dissociates in its absorption and distribution after application on the skin [165], with plasma protein binding ranging from 5.9–12.2% [178]. The organic pyrithione moiety permeates and distributes faster in the major organs compared to the inorganic zinc moiety [165]. This is important because it implicates pyrithione and its metabolites as the primary mediators of systemic toxicity.

Hepatic metabolism and subsequent elimination of pyrithione has been studied in rats, rabbits, monkeys, and dogs after oral dosing [179,180]. While several metabolites and intermediates have been identified, the major metabolites include 2-methanesulfonylpyridine (MSP) [179] and S-glucuronide conjugates of 2-pyridinethiol and 2-pyridinethiol-1-oxide (SG). Renal and biliary excretion occurs for both, of which the biliary route and faecal excretion occurs at a slower rate due to enterohepatic reabsorption. More than 75% of the dose is excreted in the urine rapidly within 24 h, primarily as SG [180].

Systemic exposure to ZnPT after topical dosing has been studied in humans and a range of animal species including rat, rabbit, monkey and guinea pig (Table 4) [12,143,165,177,181,182]. Exposure can be estimated in ex vivo Franz cell studies from the amount of ZnPT that permeates into the receptor phase or can also be investigated in vivo with urine or blood serum analysis. For human studies, urine sampling can reliably be used since more than 90% of the absorbed dose is excreted renally [12]. The amount of ZnPT exposure depends on a range of factors including the skin type and condition (intact, tape stripped, or artificial sebum supplemented), dosage amount, frequency (single or repeated), formulation (simple aqueous dispersion or as shampoo) and length of exposure. There are important species dependent variations in permeability, with rabbit skin showing the highest permeability, followed by rat and guinea pig [182].

ZnPT permeation in all cases remains relatively very low, typically representing less than 0.05% of the initial applied dose. Absorption can be increased by addition of surfactant [143,181], by removal of stratum corneum [143] and for aqueous formulations by the presence of artificial sebum [181]. Studies in both SD and healthy skin show no effect of disease on percutaneous absorption [12]. Increasing the contact time, ZnPT dose, and number of applications does increase exposure, however a steady state is reported to be reached after 4 days daily application [12]. The highest in vivo human exposure of 4.38 μg/kg/day occurs with daily shampoo applications (ZnPT 2%) and use of leave-on tonic (ZnPT 0.25%) for 4 days. This is in close agreement to the human dose level (4.4 μg/kg) projected in earlier studies based on rat exposure data to a 1% ZnPT formulation [182]. In both cases, demonstrating that human exposure is well below (on the order of 100-fold lower) than toxicity thresholds reported, for instance 0.5 mg/kg/day as the no effect level in rats orally fed ZnPT [144].

Recognizing that toxicological exposure studies often represent exaggerated use sceneries (i.e., daily at fixed, typically high doses), computer modelling approaches are making an in-roads to capture realistic ZnPT exposure for human risk assessment. A probabilistic model has reported on the aggregate exposure of ZnPT [183], taking into account information about different product usage patterns of the population (e.g., users that apply different amounts, at different frequencies and from different product sources). In this model, exposure was calculated as 0.01–1.29 μg/kg/day for the top 95th percentile of users, which is again well below threshold for adverse systemic effects. In separate work, physiologically based pharmacokinetic models have been validated for both oral [184] and dermal delivery [185] of ZnPT in the rat, capable of dosimetry predictions for hindlimb weakness. Further research is needed to extrapolate this to humans, however this nevertheless represents an important step forward for the use of computer modelling approaches in safety assessment, which should result in less reliance on animal use in the future.

## 7. Future Directions and Conclusions

Observations on seborrheic dermatitis, or at least conditions that mirror its symptomology, have been made since ancient times. This notably includes Celsus and Galen from the 2nd century AD, who postulated on the origins of skin squames [186]. It was not however until the nineteenth century that an association with yeast was made by Louis-Charles Malassez [52]. Significant advances have since been made in the development of treatment options in SD, including the formulation of ZnPT based shampoos. It should also be recognized that ZnPT is being explored in other applications (Table 5), such as treating atopic dermatitis and fungal infections, which may prove to have significant clinical impact in the future.

A question central to assessing therapeutic performance is whether ZnPT reaches the target sites in SD at appropriate anti-fungal concentrations. Here, we show an average MIC of 10–15 ppm derived from published in vitro broth microdilution studies against *Malassezia globosa* and *restricta*. A further goal of targeted delivery with ZnPT is to maximize deposition and persistence on the scalp surface and within the follicles, while minimizing skin absorption which presents a risk for localized and systemic toxicity. A range of skin imaging methods have demonstrated delivery to the skin surface and uppermost portion of the hair follicles. ZnPT faces the unique challenge of achieving this targeted delivery to the scalp in a complex wash-off formulation system. Quantitative dose-depth information would be particularly useful for a more accurate assessment of ZnPT delivery, particularly to the follicles, in addition to data on yeast load in these recessed micro-environments which play an important role in SD pathogenesis.

Further formulation optimization of ZnPT products is likely to focus on improving follicular delivery and may explore strategies that have shown success for the targeting of other compounds into the follicles, including modification of particle shape and the use of functionalized surface coatings [205,206]. Targeted delivery, therapeutic efficacy and safety could then be assessed with the complementary techniques that have been examined here.

## Figures and Tables

**Table 1:** Comparison of skin imaging methods for assessing zinc pyrithione topical delivery.

Imaging Method	Advantages	Disadvantages	Key Findings
**Autoradiography** **[126,127]**	Chemical specificity and sensitivity Acquisition of both qualitative and quantitative data	Radiolabelling can modify chemical properties important for delivery Expensive Requires careful handling and preparation Safety (exposure to radiation)	Localisation of ZnPT around hair follicle opening and no penetration into dermal layer [126] Lateral micro-transport is formulation dependent [127]
**Confocal Microscopy [56,115]**	High resolution Ability to optically section skin Pseudo-quantification of delivery Non-invasive, therefore ideal to be performed in vivo	Resolution drops after approximately 150–200 µm through tissue Low chemical specificity in reflectance mode	Follicular delivery is formulation dependant, with signal detection greatest in upper infundibulum (upper 0–40 µm) [115]
**Raman Microscopy** **[128,129]**	Chemical specificity Ability to optically section skin, therefore ideal for in vivo use	Optical sectioning limited to maximum 100 µm depth Spectral interpretation can be difficult	Follicular delivery limited to upper follicle (20 µm) in porcine skin. Plate morphology of particles observable on scalp surface.
**Scanning Electron Microscopy** **(SEM) [130,131]**	High resolution Enables assessment of particle morphology Combined with X-ray spectroscopy to enable chemical specificity	Expensive Sample preparation Time consuming Only suitable for ex vivo tissue or indirect tape strips	2 µm plate morphology of particles on scalp surface [130]
**Fluorescent Lifetime Imaging Microscopy (FLIM) [131]**	Chemical specificity and sensitivity High resolution Ability to optically section skin Non-invasive technique can be performed in vivo	Expensive Acquisition time for sufficient photon count to produce image (times are improving) Histological tissue sectioning may not provide representative view of delivery	Delivery of ZnPT in aqueous suspension at up to 200 µm in hair follicle.

**Table 2:** Quantification of zinc pyrithione delivery to skin.

Analytic Method	Skin	Formulation	Dose	Exposure Time	Limit of Detection/Specific Activity	Collection Method	Skin Delivery		Ref
							Scalp Surface Delivery	Follicular Delivery	
**Ultra-high-performance liquid chromatography–tandem mass spectrometry (UHPLC–MS/MS)**	Human scalp in vivo	ZnPT 1% Climbazole 0.5% shampoo	Not stated		1 ng/mL	Cyanoacrylate glue biopsy with in-house cutting device to isolate follicle head	2770 ± 2540 ng/cm^2^	11 ± 9 ng/cm^2^	[128,145]
**High performance liquid chromatography tandem mass spectrometry (HPLC-MS/MS)**	Human scalp in vivo	1% ZnPT shampoo	Not stated		1.0 µg/mL	Hair pluck	-	0.6 ± 0.053 ng/ follicle	[115,141]
						Cyanoacrylate glue biopsy	-	22.5 ± 3.6 ng/follicle	
						Scrub solution extraction	1360 ng/cm^2^		
**X-ray fluorescence spectroscopy (XRF)**	Artificial VitroSkin™	ZnPT 1% shampoo	100 µL/cm^2^	30 sec	Not reported	Whole skin processing	11,100 ± 1590 ng/cm^2^		[142]
		ZnPT 1% Climbazole 0.5% shampoo					14.83 ± 1.81 ug/cm2		
**[^14^C]ZnPT radiolabelling and scintillation counting**	Human skin split thickness ex vivo	1% aqueous ZnPT in CMC & Darvan	10 µL/cm^2^	24 h	3.09 mCi/mmol	Tape stripping (20 times)	0.55 ug/cm^2^		[12]
	Rat skin split thickness ex vivo						1.55 ug/cm^2^		
		48% aqueous ZnPT in CMC & Darvan					48.94 ug/cm^2^		
	Monkey scalp in vivo	0.1% ZnPT in 0.1% surfactant	40 µL/cm^2^	3 h	60mCi/g	Tape stripping (15 times)	0.0035 ug/cm^2^ (0.009%)		[143]
				72 h			0.045 ug/cm^2^ (0.115%)		
	Human scalp in vivo	1% ZnPT shampoo + 0.1% tonic (2 applications)	10 g each application	4 days	3.09mCi/mmol	Tape stripping (frequency not reported)	0.51 ug/cm^2^		[12]
		2% ZnPT shampoo + 0.1% tonic (2 applications) + 0.25% tonic (1 application)					1.39 ug/cm^2^		
		2% ZnPT + 0.25% tonic (3 applications)					1.92 ug/cm^2^		
**[S^35^]ZnPT radiolabelling and scintillation counting**	Human forearm in vivo	1% ZnPT shampoo	200 µL/cm^2^	5 min	0.6 µg/g	Radioactivity measured directly over skin	3 µg/cm^2^ (1.4% of applied)		[144]

**Table 3:** Exposure of zinc pyrithione to viable skin.

Skin	Formulation	Applied Dose	Viable Skin Delivery	Exposure Time	Ref
**Ex vivo split thickness rat skin**	ZnPT 48%, Darvan, carboxymethylcellulose, water	10 µL/cm^2^	118.64 µg/cm^2^	8 h	[12]
	ZnPT 1%, Darvan, carboxymethylcellulose, water		6.68 µg/cm^2^		
**Ex vivo split thickness human skin**			0.75 µg/cm^2^		
**In vivo monkey scalp skin**	0.1% ZnPT suspension with surfactant (triethanolamine alkyl sulfate)	0.4 mL/10cm^2^	0.085% (0.47 µg/cm^2^)	3 h	[143]
			0.090% (0.5 µg/cm^2^)	72 h	

Viable skin delivery = epidermis (after stratum corneum removal with tape stripping) + dermis.

**Table 4:** Human and animal systemic exposure values for zinc pyrithione applied in various formulations.

Skin	Formulation	Applied Dose	Absorbed Dose (Cumulative)	Exposure Time	Ref
Ex vivo split thickness rat skin	ZnPT 48% *w*/*v*, Darvan, carboxymethylcellulose, water	10 µL/cm^2^	0.13% (equiv. 7.01 µg/cm^2^)	8 h, plus 16 h after removal from donor chamber (24 h total)	[12]
	ZnPT 1% *w*/*v*, Darvan, carboxymethylcellulose, water		1.12% (equiv. 1.13 µg/cm^2^)		
Ex vivo split thickness human skin			0.02% (equiv 0.02 µg/cm^2^)		
Ex vivo split thickness human skin	1% *w*/*v* ZnPT (1% *w*/*v* carboxymethylcellulose)	5 mL/0.79cm^2^	0.047 µg/cm^2^	72 h	[181]
	1% *w*/*v* ZnPT (1% *w*/*v* body wash)		0.19 µg/cm^2^		
	1% *w*/*v* ZnPT (castor oil)		0.23 µg/cm^2^		
Ex vivo split thickness human skin, sebum supplemented	1% *w*/*v* ZnPT (1% *w*/*v* carboxymethylcellulose)	5 mL/0.79cm^2^	0.14 µg/cm^2^	72 h	[181]
	1% *w*/*v* ZnPT (1% *w*/*v* body wash)		1.1 µg/cm^2^		
	1% *w*/*v* ZnPT (castor oil)		0.28 µg/cm^2^		
In vivo rat skin	ZnPT 48% *w*/*v*, Darvan, carboxymethylcellulose, water	10 µL/cm^2^	0.19%	8 h, occlusive dressing	[12]
	ZnPT 1% *w*/*v*, Darvan, carboxymethylcellulose, water		0.85%		
In vivo rat skin	5% *w*/*v* ZnPT + 10% *w*/*v* EDTA in shampoo	Not reported	1 µg	24 h	[177]
	5% *w*/*v* ZnPT in shampoo		1 µg		
In vivo rabbit skin	1% *w*/*v* aqueous suspension	40 mg/kg (4 mL/kg)	0.5%	8 h total (4 h before rinsing)	[165]
In vivo monkey skin, abdomen	2% *w*/*v* ZnPT aqueous suspension	30 mg/kg	0.012–0.039%	3 h	[143]
			0.02%	11 days (3 applications)	
	2% *w*/*v* ZnPT suspension with surfactant (triethanolamine alkyl sulfate)	30 mg/kg	0.032%	3 h	
			0.2%	4 days (3 applications)	
In vivo monkey skin, abdomen (tape stripped)	2% *w*/*v* ZnPT suspension with surfactant (triethanolamine alkyl sulfate)	30 mg/kg	0.29%	3 h	
In vivo monkey skin, scalp	0.1% *w*/*v* ZnPT suspension with surfactant (triethanolamine alkyl sulfate)	0.4 mL/10cm^2^ (400 µg/10cm^2^)	1.19–4.39%	3 h	
			2.73–3.36%	72 h	
In vivo rat skin	1% *w*/*v* ZnPT in shampoo base	0.1 mL/7.5cm^2^	0.17 µg/cm^2^	10-min contact before rinse (24 h monitoring)	[182]
In vivo guinea pig skin		0.3 mL/22.5cm^2^	0.06 µg/cm2		
In vivo rabbit skin		1 mL/75cm^2^	0.98 µg/cm2		
In vivo human skin	ZnPT shampoo 1% *w*/*v*	10 g shampoo 4 g tonic per application	1.02 µg/kg/day	1 day	[12]
	ZnPT shampoo (1% *w*/*v*) + tonic (0.1% *w*/*v*)		1.39 µg/kg/day	1 day	
	ZnPT shampoo 1% *w*/*v*		2.76 µg/kg/day	4 days daily application	
	ZnPT shampoo (1% *w*/*v*) + tonic (0.1% *w*/*v*)		3.43 µg/kg/day	4 days daily application	
	ZnPT shampoo (2% *w*/*v*) + tonic (0.1% *w*/*v*)		4.38 µg/kg/day	4 days, 2 application	

**Table 5:** Therapeutic re-purposing of zinc pyrithione.

Use	Evidence	Comment
**Psoriasis**	ZnPT spray (0.25% *w*/*v*) used twice daily has been effective in treating psoriatic plaques in one case showing almost complete clearing over 3 weeks with no significant side effects [187]. The classic histopathologic features of psoriasis treated with ZnPT spray (0.25% *w*/*v*) in a separate case have also been reported to resolve over 2 weeks [188]. A randomised double-blind placebo control study (*n* = 60) found that 0.25% *w*/*v* ZnPT emollient cream used twice daily for 3 months led to a significant reduction in severity of indurations, erythema and scaling [189].	Results should be interpreted cautiously given the findings from early case studies [187,188] used SkinCap, a commercial product now withdrawn from the market due to reports of contamination with prescription steroid clobestol propionate [190]. A randomised double-blind study (*n* = 25) found ZnPT in similar formulation did not enhance the efficacy of clobestol propionate [191]. ZnPT for psoriasis remains controversial and further evidence is required to determine utility as an adjunct or alternative to conventional medications, which can have important side effects including immune suppression.
**Atopic dermatitis and eczema**	In atopic dermatitis and eczema *Malessezia* yeast and its metabolites have the potential to cause barrier aggravation [192]. and IgE binding allergens involved in the immune response [193]. Approximately half of the differentially expressed stratum corneum proteins from dandruff and atopic dermatitis are the same, suggestive of a common aetiology [34]. A randomised controlled trial in Chinese children (*n* = 95) showed benefits of a ZnPT ultra-mild body wash with lipids (concentration not reported) on skin microbiome diversity and atopic dermatitis score (SCORAD) when used in conjunction with 0.1% *w*/*v* hydrocortisone butyrate cream [194].	ZnPT formulated in body wash may provide adjunctive treatment to corticosteroid use for management of atopic dermatitis and eczema. ZnPT may act by altering skin levels of *Malassezia* yeast or *Staphylococcus aureus* bacteria, known to be important in aggravating atopic dermatitis [195]. Further evidence of benefit from larger randomised controlled trials is required.
**Skin antisepsis**	ZnPT exhibits gradual activity against a broad spectrum of gram negative and positive bacteria as well as RNA and DNA viruses [196] for up to three days in an expanded flora test on the forearm [197]. Concentrations up to 0.25% *w*/*v* ZnPT with alcohol were superior to other combination agents (e.g., iodine, chlorhexidine gluconate and triclosan) for persistence of antimicrobial effects [198].	ZnPT in antiseptic products has the potential for significant clinical impact, for example by reducing surgical site infections through extended anti-microbial persistence. Efficacy needs to be confirmed in randomised controlled trials and safety needs further evaluation.
**UVB induced hyperplasia**	In mice exposed to ultraviolet-B (UVB) radiation a 1% *w*/*v* ZnPT cream prevented skin thickening and normalized [199] levels of hypoxia-inducible factor-1α, which influences the keratinocyte cell cycle [200].	ZnPT could be used to prevent UVB-induced photoaging and skin cancer development, potentially as an additive to sunscreens. Further work is needed to demonstrate human efficacy and establish therapeutic doses.
**Hair loss**	Sub-clinical inflammation of the scalp [201] and *Malessezia* yeast metabolites [65] are believed to damage the hair shaft and promote hair loss [202,203]. In a 6-month clinical trial 1% *w*/*v* ZnPT shampoo caused a significant increase in total visible hair count in males (*n* = 200), with similar performance to 5% *w*/*v* minoxidil topical solution [204].	ZnPT may promote general hair and scalp health [81], for example by providing a source of zinc and reducing inflammation, which could play a role in preventing, delaying or improving symptoms of hair loss.

## Data Availability

Not applicable.
